# Lipolysis in Health and Disease: Pathways, Regulation, and Metabolic Consequences

**DOI:** 10.1007/s11892-026-01624-7

**Published:** 2026-04-10

**Authors:** Courtney L. Bordelon, Jacqueline M. Stephens

**Affiliations:** 1 Pennington Biomedical Research Center, Louisiana State University, 6400 Perkins Road, Baton Rouge, LA 70808 USA; 2 Department of Biological Sciences, Louisiana State University, Baton Rouge, LA 70803 USA

**Keywords:** Adipocyte, Lipolysis, Metabolic disease

## Abstract

**Purpose of Review:**

Lipolysis regulates lipid distribution, energy availability, and metabolic homeostasis in organisms that store triacylglycerols. In multicellular organisms, adipose tissue evolved as a specialized organ that centralizes lipid storage and release, buffers nutrient fluctuations, and protects non-adipose tissues from lipid overload. Dysregulated adipocyte lipolysis occurs across diverse metabolic conditions, including obesity, diabetes, lipodystrophy, and inflammatory states. This review synthesizes evidence that defective suppression of basal lipolysis and impaired responsiveness to physiological stimuli disrupt lipid partitioning, promote insulin resistance, and drive ectopic lipid accumulation.

**Recent Findings:**

Research shows that metabolic dysfunction arises in both obese and non-obese settings and correlates more closely with impaired control of fatty acid flux than with adiposity itself. Higher expression of lipolytic receptors, including those targeted by weight-loss medications, together with genes that regulate lipolysis through insulin signaling, is associated with greater weight loss and improved systemic metabolic health.

**Summary:**

In this review, we highlight canonical and noncanonical mechanisms governing lipolytic regulation and contrast pathological lipolysis with the tightly controlled lipolysis observed during weight loss. Together, these findings reframe lipolysis as a central determinant of metabolic health and a therapeutic target when precisely regulated.

## Introduction

All organisms that store triacylglycerols (TGs) rely on lipolysis to release fatty acids and glycerol for energy use and to regulate lipid distribution between tissues. In simpler organisms, lipid droplets are present across many cell types and lipolysis operates locally within individual cells [1]. As some multicellular organisms increased in size and complexity and adapted to repeated feeding–fasting cycles, adipose tissue emerged as a specialized organ that centralizes lipid storage and release. In this role, adipose tissue buffers fluctuations in nutrient availability, protects non-adipose tissues from ectopic lipid accumulation, and supports systemic metabolic stability [2]. Beyond energy storage, adipose tissue functions as a dynamic endocrine organ whose regulation influences the integrity of multiple tissues and overall systemic metabolic health. Disruption of adipocyte function, particularly lipolysis, is therefore associated with a wide range of pathological states [3].

Dysregulated lipolysis is observed across diverse metabolic conditions, including obesity, diabetes, rare genetic disorders, and inflammatory or immune-mediated diseases. In diabetes and metabolically unhealthy obesity, defective suppression of lipolysis in the fed state and inadequate stimulation during fasting impair the temporal control of fatty acid flux, contributing to insulin resistance and broader features of metabolic syndrome [4]. Rare disorders such as congenital generalized and partial lipodystrophy further illustrate the consequences of uncontrolled lipolysis. Despite reduced or absent adipose tissue, some lipodystrophic patients exhibit excessive lipid flux, severe insulin resistance, and fatty liver disease [5, 6]. Conversely, neutral lipid storage diseases are characterized by impaired lipolysis and lipid accumulation in tissues such as skeletal muscle yet typically lack the systemic features of metabolic syndrome [7]. Inflammatory and immune-mediated states can also disrupt lipolytic homeostasis; during sepsis, cancer, and critical illness, sustained cytokine signaling and sympathetic activation markedly increase lipolysis, promoting metabolic instability and tissue dysfunction [8–10].

A unifying feature across these conditions is dysregulated fatty acid flux rather than adiposity per se. Metabolic dysfunction arises in both obese and non-obese states, and in many cases impaired control of lipolysis contributes directly to insulin resistance independent of total fat mass [8–11]. Despite this, insulin resistance and diseases such as diabetes are frequently conflated with obesity, particularly in discussions of lipid metabolism. Lipolysis, for example, has been portrayed both as a causal driver of insulin resistance and as a desirable therapeutic target for weight loss [12–14]. This oversimplified framing reinforces the notion that increased fat mass is inherently pathogenic, overlooking a critical distinction: obesity often exists without uncontrolled lipolysis, and dysregulated lipolysis can occur in the absence of obesity.

Separating body weight from lipid homeostasis is therefore essential for understanding metabolic disease and identifying effective therapeutic strategies. This review aims to reframe lipolytic homeostasis as a key mechanism distinguishing obesity from metabolic dysfunction, with a particular emphasis on insulin sensitivity. We deliberately focus on the regulation of lipolysis and the disorders in which it is perturbed, rather than on adiposity itself. Our goal is to provide a conceptual framework in which lipolysis is understood as a process that can exacerbate metabolic disease when dysregulated, but when tightly controlled, can also be leveraged to improve metabolic health independent of body fat mass.

### Regulators of the Enzymatic Cascade

Lipolytic flux is determined less by enzyme abundance than by tightly coordinated post-translational control, spatial organization, tissue specificity, and opposing hormonal signals. The canonical lipolytic pathway consists of three enzymes: adipose triglyceride lipase (ATGL), hormone-sensitive lipase (HSL), and monoacylglycerol lipase (MGL). Despite being identified most recently, ATGL catalyzes the initial and quantitatively dominant step, hydrolyzing triacylglycerols to diacylglycerols and accounting for most triglyceride hydrolysis in adipose tissue [15, 16]. Although ATGL mRNA expression is upregulated by fasting, glucocorticoids, Peroxisome Proliferator-Activated Receptor (PPAR) agonists, and Sirtuin 1–Forkhead Box 01 (SIRT1–FoxO1) signaling, and suppressed by insulin, feeding, and mechanistic Target of Rapamycin Complex 1 (mTORC1) activity, transcript levels do not reliably predict lipolytic activity [17]. β-Adrenergic stimulation and inflammatory cues such as TNF-α can suppress ATGL mRNA while increasing lipolysis, underscoring the primacy of post-translational regulation [18].

ATGL activity is enhanced by phosphorylation at serine 406 via AMP-activated protein kinase (AMPK) and by its obligate coactivator comparative gene identification 58 (CGI-58) [19, 20]. In the basal state, perilipin-1 coats lipid droplets and sequesters CGI-58, preventing ATGL activation. β-Adrenergic signaling activates protein kinase A (PKA), which phosphorylates perilipin-1 at multiple residues, including serine 517, releasing CGI-58 and enabling ATGL activation [21]. Disruption of this spatial control, as seen with pathogenic perilipin-1 mutations, causes unrestrained lipolysis, partial lipodystrophy, hypertriglyceridemia, and insulin resistance [22]. Additional layers of regulation emphasize tissue specificity: the ATGL inhibitor G0S2 is suppressed by fasting in adipose tissue but induced in liver [23, 24]. Other liver and adipose tissue differences include, how ATGL and HSL account for over 90% of lipolytic activity in white adipose tissue, whereas in fasted liver ATGL contributes less than half of triglyceride hydrolase activity; accordingly, ATGL deficiency causes hepatic steatosis without impairing VLDL secretion, indicating compensatory lipase activity [16, 25].

HSL catalyzes the rate-limiting step in diacylglycerol hydrolysis and is likewise regulated predominantly by post-translational and spatial mechanisms. HSL is activated by β-adrenergic signaling and inhibited by insulin, with phosphorylation by PKA at serine residues 660 and 663 serving as key activating events. Additional modulation occurs through AMPK, Extracellular signal-regulated kinase 1/2 (ERK), Ca2⁺/calmodulin-dependent kinase, and glycogen synthase kinase pathways [26, 27]. Full activation requires translocation of HSL to the lipid droplet, mediated by interaction with phosphorylated perilipin-1; coregulators such as Receptor interacting protein of 140 kDa (RIP-140) facilitate this process by promoting perilipin-1–HSL interactions [28]. Monoacylglycerol lipase (MGL) completes the cascade by hydrolyzing monoacylglycerols. MGL is ubiquitously expressed, localizes to membranes, cytoplasm, and lipid droplets, and is most abundant in adipose tissue. Genetic disruption of MGL impairs lipolysis and results in monoacylglycerol accumulation across tissues, confirming its essential role in lipid turnover [29].

**Fig. 1:**
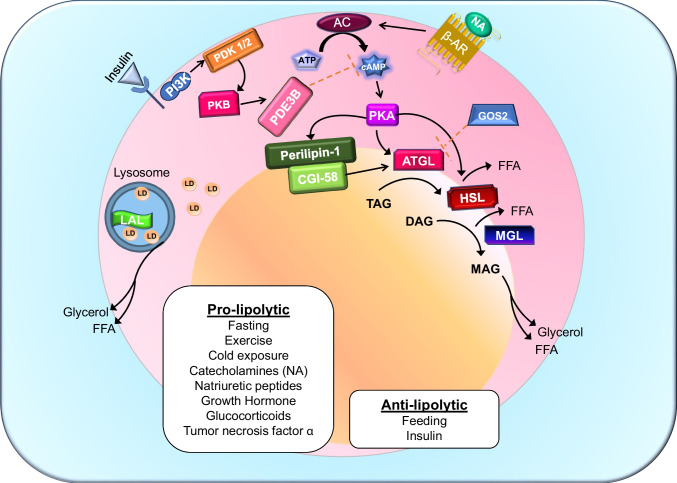
Canonical and lysosomal regulation of adipocyte lipolysis. Pro-lipolytic stimuli including fasting, exercise, cold exposure, catecholamines (noradrenaline, NA), glucocorticoids, growth hormone, and tumor necrosis factor-α activate β-adrenergic receptors (β-AR), stimulating adenylyl cyclase (AC) to increase cAMP production and activate protein kinase A (PKA). PKA phosphorylates lipid droplet-associated proteins such as perilipin-1, promoting release of CGI-58 and activation of adipose triglyceride lipase (ATGL). Sequential hydrolysis of triacylglycerol (TAG) to diacylglycerol (DAG), monoacylglycerol (MAG), and ultimately glycerol and free fatty acids (FFAs) is mediated by ATGL, hormone-sensitive lipase (HSL), and monoacylglycerol lipase (MGL), respectively. Insulin and feeding exert anti-lipolytic control primarily through activation of phosphodiesterase 3B (PDE3B), reducing cAMP levels and attenuating PKA signaling, thereby preserving lipid droplet integrity and limiting fatty acid release. Lysosomal acid lipase (LAL) contributes to lipid mobilization via lipophagy, providing an additional layer of regulation independent of the canonical cytosolic lipase cascade. Figure adapted from review [33]. Abbreviations: β-AR, β-adrenergic receptor; NA, noradrenaline; AC, adenylyl cyclase; PKA, protein kinase A; PDE3B, phosphodiesterase 3B; ATGL, adipocyte triglyceride lipase; HSL, hormone-sensitive lipase; MGL, monoacylglycerol lipase; LAL, lysosomal acid lipase; TAG, triacylglycerol; DAG, diacylglycerol; MAG, monoacylglycerol; FFA, free fatty acid; PI3K, Phosphoinositide 3-kinase, GOS2, G₀/G₁ Switch Gene 2; PDK1/2, 3-phosphoinositide-dependent protein kinase; PKB, Protein Kinase B

At the systemic level, insulin is the dominant antilipolytic hormone. At low physiological concentrations, insulin suppresses lipolysis through activation of phosphodiesterase 3B (PDE3B), reducing cAMP levels and attenuating PKA signaling, thereby preserving lipid droplet integrity [30]. Notably, insulin’s antilipolytic effects are more sensitive than its actions on glucose uptake, highlighting the priority of lipid containment in the fed state [31]. Insulin signaling within the central nervous system further restrains lipolysis by reducing sympathetic outflow to adipose tissue [32]. In contrast, catecholamines and glucagon stimulate lipolysis via cAMP–PKA signaling during fasting, cold exposure, exercise, and acute stress. These opposing inputs generate a pulsatile pattern of lipolysis that allows rapid, reversible mobilization of energy while minimizing lipid spillover and preserving metabolic flexibility. The core enzymes and regulatory nodes governing this cascade are summarized in Fig. 1.

### Beyond the Canonical Cascade

While the ATGL–HSL–MGL cascade defines canonical lipolysis, lipolytic flux and lipid remodeling are shaped by additional enzymatic pathways that operate across subcellular compartments and tissues. Lipid droplet–associated enzymes, redundant triglyceride hydrolases in non-adipose tissues, and lysosomal or autophagic lipolytic pathways together establish basal lipolytic tone and expand the regulatory capacity of lipid mobilization.

Several enzymes act directly at the lipid droplet alongside ATGL and HSL. PNPLA3, a member of the same protein family as ATGL, modulates lipid droplet remodeling and functions primarily in the fed state [34]. The common I148M variant (rs738409) exhibits reduced triglyceride hydrolase activity and increased retention at the lipid droplet surface, impairing lipid turnover and potentially restricting ATGL access to stored triglycerides [35]. This defect promotes hepatic triglyceride accumulation and increases susceptibility to steatosis and fibrosis, often independent of overall adiposity or elevated circulating fatty acids, highlighting how local lipid handling can drive tissue-specific pathology [35].

Other noncanonical lipases contribute to lipid signaling and metabolism outside adipose tissue. ABHD6 (α/β-hydrolase domain–containing protein 6) is a serine hydrolase localized primarily to the endoplasmic reticulum and, to a lesser extent, lipid droplets. Although it partially overlaps with MGL in hydrolyzing monoacylglycerols, ABHD6 plays a more prominent role in non-adipose tissues, particularly the liver and central nervous system [36]. By regulating monoacylglycerol pools—including the endocannabinoid 2-arachidonoylglycerol (2-AG)—ABHD6 links lipid metabolism to signaling pathways [37]. In metabolic tissues ABHD6 inhibition improves insulin sensitivity and hepatic lipid accumulation in diet-induced obesity models, underscoring its role in coupling lipid turnover to metabolic regulation [38].

Lipolytic dominance also varies markedly by tissue. In the liver, triglyceride hydrolysis relies on enzymes beyond the canonical cascade, including carboxylesterases such as CES1. CES1 contributes to hepatic triglyceride hydrolysis and can partially compensate for ATGL deficiency [39, 40]. Unlike ATGL, which operates at the cytosolic surface of lipid droplets and is tightly regulated by β-adrenergic and insulin signaling, CES1 functions within the endoplasmic reticulum. There, it participates in the mobilization of triglycerides destined for very-low-density lipoprotein (VLDL) assembly and secretion, positioning CES1 at the interface between intracellular lipid mobilization and lipoprotein production [41].

Not all lipid mobilization occurs at neutral pH. LIPA encodes lysosomal acid lipase (LAL), which hydrolyzes cholesteryl esters and triacylglycerols within lysosomes as part of the lipophagic pathway. Unlike ATGL and HSL, which act on cytosolic lipid droplets, LAL functions downstream of autophagy following delivery of lipid droplets to lysosomes [42]. Adipocyte LIPA expression and activity increase during prolonged fasting, cold exposure, and β-adrenergic stimulation; inhibition of LAL blunts fasting-induced rises in plasma free fatty acids and impairs thermogenesis and oxygen consumption, demonstrating its importance for lipid mobilization [43]. In hepatocytes, LAL-driven lipophagic flux supplies fatty acids for oxidation and integrates lipid turnover with cholesterol sensing and trafficking pathways [44]. Because LAL activity is regulated by autophagic flux and lysosomal biogenesis programs, including MiT/TFE family transcription factors, this pathway is highly sensitive to nutrient state and energy stress [45]. Loss of LIPA causes Wolman disease or cholesteryl ester storage disease, characterized by severe hepatic lipid accumulation and systemic metabolic dysfunction [46]. Lysosomal lipolysis remains less characterized, and its relative contribution—whether complementary to the canonical pathway or essential during high energy demand—has yet to be defined.

### Obesity and Lipolysis

Obesity is metabolically heterogeneous. As summarized in Table 1, metabolic outcomes across diverse disease states are more accurately organized along a spectrum defined by lipolytic regulation rather than adiposity alone. Both low- and high-fat states can exhibit pathology when lipolytic control is lost. Although increased fat mass defines obesity, only a subset of individuals with obesity display dysregulated lipolysis [4]. Individuals who retain normal metabolic parameters despite obesity are classified as metabolically healthy obese (MHO), a phenotype observed in approximately 10–30% of obese populations and therefore not rare. Diagnostic criteria for MHO include a BMI ≥ 30 kg/m^2^, fasting triglycerides ≤ 1.7 mmol/L, HDL cholesterol > 1.0 mmol/L in men or > 1.3 mmol/L in women, and blood pressure ≤ 130/85 mmHg [4].

**Table 1:** Human diseases and physiological states organized by direction of lipolytic activity and adiposity

Condition/State	Lipolytic activity	Adiposity	Fatty acid flux to peripheral tissues	Insulin sensitivity	Reference
Metabolically Healthy Lean	↔	↔	Matched to oxidation	↔	[47]
Prolonged Fasting	↑	↓	Matched to oxidation	↑	[48]
Metabolically Healthy Obesity (MHO)	↔	↑	Buffered within adipose	↔	[4]
Early Overnutrition	↑ (mild)	↑	Beginning to exceed oxidation	↓ (mild)	[4]
Obesity with Insulin Resistance	↑	↑	Chronic spillover	↓	[4]
Nonalcoholic Steatohepatitis (NASH)	↑	↔/↑	Persistent hepatic overload	↓	[49]
Type 2 Diabetes	↑	↔/↑	Chronic spillover	↓↓	[47]
Type 1 Diabetes Mellitus	↑↑	↓/↔	Unrestrained systemic release	↓↓	[47]
Congenital Generalized Lipodystrophy	↑	↓↓	Unbuffered spillover	↓↓	[5]
Partial Lipodystrophy (including PLIN1 mutations)	↑	↓	Unbuffered spillover	↓↓	[6]
HIV-associated Lipodystrophy	↑	↓	Spillover to liver/muscle	↓	[50]
Cachexia	↑↑	↓↓	Massive systemic release	↓	[9]
Sepsis/Acute Critical Illness	↑↑	↔/↓	Acute FA surge	↓	[51]
Rheumatoid Arthritis	↑	↔	Inflammation-driven spillover	↓	[52]
Cushing’s Syndrome	↑	↑	Increased visceral flux	↓	[53]
Neutral Lipid Storage Disease (NLSD)	↓↓	↑	Reduced FA mobilization	↔	[7]
Wolman Disease/Cholesteryl Ester Storage Disease (LIPA deficiency)	↓↓	↔	Defective intracellular clearance	↔/↓	[46]
PNPLA3 I148M–associated Steatosis/Fibrosis	↓	↔	Hepatic retention/remodeling	↔/↓	[35]

Conditions are arranged to illustrate how alterations in adipocyte lipolysis occur across diverse metabolic, inflammatory, endocrine, and genetic disorders. Arrows denote relative changes compared to physiologic baseline: ↑ increased, ↑↑ markedly increased, ↓ decreased, ↓↓ markedly decreased, and ↔ no major change. Notably, both increased and decreased lipolysis are observed in states of low, normal, and high adiposity, reinforcing that metabolic dysfunction aligns more closely with the regulation and suppressibility of fatty acid flux than with absolute fat mass. Hyperlipolytic states driven by insulin deficiency, inflammation, or hormonal excess often associate with insulin resistance despite reduced adiposity. Abbreviations: *MHO* metabolically healthy obesity, *NASH* nonalcoholic steatohepatitis, *HIV* human immunodeficiency virus, *NLSD* neutral lipid storage disease, *LIPA* lysosomal acid lipase gene, *PNPLA3* patatin-like phospholipase domain–containing protein 3, *FA* fatty acid

For many individuals, however, metabolic health in obesity is transient. One of the earliest abnormalities preceding overt metabolic disease is impaired insulin-mediated suppression of lipolysis, which frequently emerges before detectable hyperglycemia [54]. In this state, basal lipolytic tone remains inappropriately elevated despite circulating insulin, resulting in chronic low-level fatty acid release [55]. Concurrently, obesity-associated inflammation promotes catecholamine resistance through downregulation of β-adrenergic signaling components, impairing stimulated lipolysis [56]. Thus, adipocytes in metabolic dysfunction often display a paradoxical phenotype characterized by inadequate suppression of basal lipolysis alongside impaired execution of stimulated lipolysis. These defects reflect a loss of regulatory precision rather than a simple excess of fat mass.

Excess fatty acid flux contributes to insulin resistance in part through the accumulation of bioactive lipid intermediates. Elevated free fatty acids (FFAs) impair insulin signaling, disrupt mitochondrial function, and activate inflammatory pathways [57]. In liver and skeletal muscle, accumulation of diacylglycerols and ceramides interferes with insulin signaling cascades [58]. In pancreatic β-cells, chronic exposure to fatty acids—particularly palmitate and ceramides—induces endoplasmic reticulum stress, oxidative injury, inflammation, and secretory dysfunction, accelerating β-cell failure [59]. Insulin resistance correlates strongly with circulating non-esterified fatty acids and bioactive lipid intermediates [60], reinforcing a self-sustaining cycle of dysregulated lipolysis and metabolic decline.

The anatomical source of lipolysis further modulates metabolic risk. Visceral adipose tissue is particularly pathogenic due to higher basal lipolytic activity, reduced insulin sensitivity, increased inflammatory signaling, and direct portal drainage to the liver. This portal delivery of fatty acids promotes hepatic gluconeogenesis, triglyceride synthesis, and very-low-density lipoprotein (VLDL) secretion, amplifying metabolic dysfunction more acutely than subcutaneous adipose tissue [61]. Experimental activation of adipocyte lipolysis acutely increases circulating FFAs to approximately 1.5 mM within 30 min, followed by a comparable rise in circulating triglycerides. In contrast, hepatic triglyceride levels peak later, approximately three hours after stimulation, and remain elevated for over 12 h despite normalization of circulating lipids. When adipocyte fatty acid release is impaired, as in mice lacking ATGL specifically in adipocytes, this hepatic triglyceride accumulation does not occur [62]. These findings are particularly relevant given the rising prevalence of metabolic function-associated fatty liver disease (MAFLD).

Importantly, triglycerides themselves are metabolically inert storage molecules rather than signaling toxins. Esterification of fatty acids into triglycerides neutralizes their detergent-like properties and limits conversion into bioactive lipid intermediates. Lipid storage therefore functions as a protective buffering system that accommodates fluctuations in nutrient availability. This buffering capacity is mediated by lipid droplets across multiple tissues, including adipose tissue, liver, and skeletal muscle [2, 3]. Lipid droplets are dynamic organelles equipped with regulatory proteins and enzymatic machinery that govern lipid sequestration and mobilization. Effective lipid containment within adipose tissue shields liver, muscle, and pancreas from lipid overload; it is the failure of this containment system—rather than triglyceride accumulation per se—that poses the primary metabolic threat associated with insulin resistance [2, 3].

Multiple lines of evidence challenge a causal relationship between adiposity and insulin resistance. The “athlete’s paradox” demonstrates that endurance-trained individuals exhibit elevated intramyocellular triglyceride content while remaining highly insulin sensitive. Subsequent studies show that exercise in older obese adults improves insulin sensitivity alongside favorable lipid partitioning, independent of weight loss [63]. The primacy of lipid handling over adiposity is further supported by transplantation studies in which subcutaneous adipose tissue from exercised mice confers marked improvements in insulin sensitivity when transplanted into sedentary, high-fat diet–fed obese mice [64]. Similarly, ATGL-, HSL-, and MGL-deficient mice accumulate large triglyceride stores equivalent to wild-type mice on a high-fat diet, yet maintain preserved or even improved insulin sensitivity, likely due to reduced systemic fatty acid exposure [29, 65, 66]. In humans, neutral lipid storage diseases—including neutral lipid storage disease with myopathy and with ichthyosis—feature extensive triglyceride accumulation across tissues without a corresponding requirement for diabetes or severe insulin resistance [7]. Collectively, these examples demonstrate that triglyceride burden alone is insufficient to drive insulin resistance.

Conversely, diminished metabolic health does not require excessive adiposity. Compared with European populations, Asian cohorts with type 2 diabetes develop disease at lower mean body mass index and are characterized by early β-cell dysfunction [11]. These observations further support the concept that insulin resistance is fundamentally a disorder of lipid flux regulation rather than adiposity.

Disorders marked by reduced adipose tissue mass frequently exhibit profound metabolic pathology. Congenital generalized and partial lipodystrophies, characterized by severely limited adipose storage capacity, are associated with uncontrolled fatty acid flux, ectopic lipid deposition, severe insulin resistance, and fatty liver disease [5, 6, 67]. Similarly, pathogenic mutations in perilipin-1 (PLIN1), which disrupt lipid droplet regulation and CGI-58 sequestration, lead to unrestrained lipolysis, partial lipodystrophy, and metabolic syndrome despite reduced or redistributed fat mass [22].

Hyperlipolytic states further dissociate adiposity from metabolic outcomes. In cancer cachexia and experimental sepsis, adipose tissue mass declines while lipolysis remains chronically elevated due to inflammatory cytokine signaling and heightened sympathetic activation. Sustained fatty acid release under these conditions contributes to systemic insulin resistance, hepatic lipid overload, and multi-organ dysfunction, despite low or declining adiposity [8, 9].

Sex-specific differences in lipid handling also influence metabolic vulnerability. Despite higher rates of overall adiposity in women, men frequently exhibit greater metabolic risk per unit of fat mass, suggesting fundamental differences in lipid partitioning and lipolytic regulation. Notably, women exhibit approximately a 50% reduction in lipolytic sensitivity to catecholamines [68]. These differences are unlikely to be driven solely by sex hormones, as neither menstrual status nor gender-affirming hormone therapy significantly alters catecholamine-stimulated lipolytic rates [68].

Together, these observations demonstrate that metabolic outcomes correlate more closely with the regulation and coordination of lipid flux than with absolute fat mass. Both insufficient and excessive lipolysis can be pathological, whereas appropriately timed, suppressible lipolysis supports metabolic health. Across diverse physiological and pathological contexts, the unifying determinant is not adiposity itself, but the precision of lipolytic control. Representative genetic models with altered lipolytic capacity and their metabolic phenotypes are summarized in Table 2, further illustrating the dissociation between triglyceride storage and insulin sensitivity.

**Table 2:** Representative genetic and pathophysiological mice models with altered lipolytic capacity demonstrate dissociation between triglyceride storage and insulin sensitivity

Gene/protein affected	Lipolysis	Adiposity	Insulin sensitivity/metabolic outcome	Reference
Systemic ATGL KO	↓	↑	Modestly increased insulin sensitivity; defective cold adaptation; cardiomyopathy develops	[65]
Adipocyte-specific ATGL KO	↓	↔/↑	Improved insulin sensitivity, equivalent or increased obesity (HFD)	[19, 69]
Adipocyte-specific ATGL overexpression	↔/↑	↓	Resistant to obesity (HFD), increased EE, increased lipolysis paired with increased oxidation, improved insulin sensitivity	[70]
Hepatocyte-specific ATGL KO	↔	↔	Hepatic TG accumulation, normal VLDL secretion, insulin sensitivity	[25]
Systemic HSL KO	↓ (mild)	↔/↑	Increased insulin sensitivity; equivalent body mass, with hypertrophic adipocytes; initial increase in insulin sensitivity (3 m), followed by lipodystrophy-induced insulin resistance (8 m)	[71, 72]
Adipocyte-specific HSL KO	↓	↔/↑	Hepatic steatosis; tissue-specific increases in adiposity, initial increase in insulin sensitivity (3 m), followed by lipodystrophy-induced insulin resistance (8 m)	[72]
Hepatocyte-specific HSL KO	↔	↔/↑	No hepatic steatosis; tissue-specific increases in adiposity	[72]
Adipocyte-specific ATGL and HSL KO	↓	↔/↑	Cold intolerant (fasting); increased BAT mass; liposarcoma	[73]
Systemic MGL KO	↓	↔	Altered endocannabinoid signaling; improved insulin sensitivity, equivalent adiposity (HFD)	[29]
Systemic Perilipin-1 KO	↑	↓	Severe insulin resistance, hepatic steatosis	[74]
Adipocyte-specific perilipin overexpression	↑	↓	Improved insulin sensitivity, lipolysis coupled with oxidation	[75]
Systemic CGI-58 KD	↔	↓/↑	Hepatic steatosis; increased insulin sensitivity; tissue-specific increases in adiposity	[76]
Systemic ABHD6 KO	-	-	Decreased inflammatory signaling;	[77]
Adipocyte-specific ABHD6 KO	↔	↔	Increased insulin sensitivity; equivalent adiposity (HFD); increased cold tolerance; hyperplasia phenotype	[37, 38]
Systemic G0S2 KO	↑	↓	Resistant to hepatic steatosis; increased insulin sensitivity; increased lipolysis coupled with increased oxidation	[78, 79]
Hepatocyte-specific G0S2 KO	↑	↓	Resistant to hepatic steatosis;	[78]
Hepatocyte-specific human CES1 overexpression	↑	-	Resistant to diet induced steatohepatitis	[40]
Adipocyte-specific LIPA KO	↓	↑	impaired thermogenesis and oxygen consumption; susceptible to diet induced obesity	[43]
Adipocyte-specific GIPR overexpression	↑	↓	Increased lipolysis coupled with increased energy expenditure; metabolic reprogramming	[80]
Cancer cachexia (Multiple models)	↑	↓	Severe inflammation; insulin resistance; unsustainable energy expenditure	[9]
Sepsis (Multiple models)	-	↓	Severe inflammation	[8]
Lipodystrophy (Multiple models)	↑	↓	Severe insulin resistance, ectopic lipid deposition	[67]

This table summarizes experimental models in which adipocyte lipolysis is genetically or physiologically altered, resulting in divergent effects on adiposity and systemic metabolic health. Collectively, these models illustrate that triglyceride accumulation alone does not uniformly predict insulin resistance, nor does reduced fat mass ensure metabolic protection. Instead, the regulation of fatty acid flux, its magnitude, tissue specificity, and capacity for oxidation or safe storage, emerges as a more accurate predictor of metabolic outcome. Abbreviations: *KO* knockout, *KD* knockdown, *HFD* high-fat diet, *EE* energy expenditure, *VLDL* very-low-density lipoprotein, *TG* triglyceride, *BAT* brown adipose tissue, *CGI-58* comparative gene identification-58 (also known as ABHD5), *ABHD6* α/β-hydrolase domain–containing protein 6, *G0S2* G0/G1 switch gene 2, *CES1* carboxylesterase 1, *LIPA* lysosomal acid lipase gene, *ATGL* adipose triglyceride lipase, *HSL* hormone-sensitive lipase, *MGL* monoacylglycerol lipase, ↑ increased, ↓↓ markedly decreased, ↑↑ markedly increased, ↓ decreased, ↔ no significant change (unchanged relative to reference state), – not reported or not determined

### Weight Loss and Lipolysis

In obesity, loss of lipolytic precision—manifesting as inadequate suppression of basal lipolysis and impaired responsiveness to appropriate stimulatory cues—drives chronic fatty acid spillover and metabolic decline. In contrast, conditions that preserve temporal control and suppressibility of lipolysis support metabolic health, even in the setting of substantial fat mass. Weight loss, therefore, provides a critical physiological context in which lipolysis is appropriately engaged to meet energetic demand while remaining tightly regulated. Examining lipolysis during weight reduction clarifies the distinction between adaptive lipid mobilization and the pathological lipolysis that characterizes metabolic disease.

Weight loss represents a physiological state in which lipolysis must increase to sustain negative energy balance yet remain precisely regulated. Reductions in circulating insulin relieve antilipolytic restraint, while catecholamine signaling permits lipid mobilization in a controlled and reversible manner. Critically, this increase in lipolytic activity remains suppressible; refeeding or insulin rapidly attenuates fatty acid release, and liberated fatty acids are typically matched to increased oxidation, limiting the accumulation of bioactive lipid intermediates. Adipose tissue also retains substantial re-esterification capacity, recycling a significant fraction of released fatty acids locally (55). Thus, weight loss–associated lipolysis reflects coordinated energy redistribution rather than dysregulation. Consistent with this distinction, metabolic improvements during weight reduction are commonly accompanied by reduced basal lipolytic tone. At the transcriptional level, greater weight loss correlates with increased expression of genes linked to insulin’s antilipolytic actions, reinforcing the concept that preserved lipolytic restraint—rather than maximal fat mobilization—underlies metabolic recovery [81].

Physical activity further refines lipolytic regulation independent of adiposity. After adjustment for BMI, age, sex, waist-to-hip ratio, adipocyte size, and cardiometabolic comorbidities, sedentary individuals exhibit impaired insulin-mediated suppression of circulating fatty acids compared with physically active subjects following insulin administration [82]. These observations show that physical inactivity is associated with persistent defects in lipolytic control. Importantly, lipolytic dysregulation may persist even after weight normalization. Prior obesity induces durable chromatin and transcriptional changes within adipocytes that blunt or distort responses to subsequent metabolic cues, a phenomenon often described as adipose metabolic memory [83].

The efficacy of modern anti-obesity pharmacotherapies further supports the principle that restoring lipolytic regulation—not maximizing fat mobilization—is central to metabolic improvement. Glucagon-like peptide-1 (GLP-1) receptor agonists enhance insulin sensitivity and enhance insulin’s antilipolytic effects, lowering basal lipolytic tone even as body weight declines. Although adipocytes do not express GLP-1 receptors, central GLP-1 signaling may indirectly influence lipid flux by modulating sympathetic output and coordinating lipolysis with fatty acid oxidation [84]. Multi-agonist therapies, including dual incretin agonists such as tirzepatide, further refine lipid flux by synchronizing fat mobilization with oxidation while preserving insulin sensitivity. Sustained activation of the glucose-dependent insulinotropic polypeptide receptor (GIPR) selectively enhances adipocyte lipolysis during fasting while maintaining insulin-mediated suppression in the fed state, aligning lipid mobilization with energetic demand rather than promoting pathological fatty acid spillover [60]. GIPR expression is enriched in adipocyte subpopulations associated with metabolic health, and adipocyte-specific GIPR overexpression in mice protects against diet-induced obesity and metabolic dysfunction. Notably, weight loss persists even after reduction of GIPR overexpression, implicating lasting changes in adipocyte metabolic programming [80].

## Conclusions

Obesity develops under chronic positive energy balance, yet progression to metabolic dysfunction is highly variable and strongly determined by the integrity of adipocyte lipolytic regulation. During early weight gain, adipose tissue expands through coordinated triglyceride storage supported by effective suppression of basal lipolysis in the fed state and appropriate activation during fasting. When this regulatory precision is maintained, adipocytes buffer excess energy, limit systemic lipid spillover, and protect non-adipose tissues from lipid overload, allowing adipose expansion without immediate loss of insulin sensitivity [4].

As adipocytes enlarge and adipose tissue becomes inflamed, lipolytic control erodes. One of the earliest abnormalities is impaired insulin-mediated suppression of lipolysis, leading to chronically elevated basal fatty acid release. Even modest increases in circulating non-esterified fatty acids enhance lipid delivery to liver and skeletal muscle, promoting hepatic gluconeogenesis, triglyceride synthesis, and accumulation of bioactive lipid intermediates that disrupt insulin signaling. Dysregulated lipolysis therefore often precedes overt hyperglycemia and contributes directly to systemic insulin resistance [54].

Defects in stimulated lipolysis further compound this process. Obesity-associated inflammation and adipocyte hypertrophy blunt β-adrenergic responsiveness, impairing lipid mobilization during energy demand [56]. The resulting phenotype—elevated basal lipolysis with reduced stimulated responsiveness—reflects loss of temporal coordination rather than excess fat mass alone, limiting metabolic flexibility and accelerating progression toward metabolic disease.

Re-esterification capacity represents an additional determinant of outcome. Normally, a substantial fraction of released fatty acids is recycled within adipocytes, restraining net efflux. When this buffering capacity is diminished, pathological lipid spillover can occur even without major changes in lipase expression. Variability in re-esterification efficiency, adipocyte size distribution, and depot-specific lipolytic responsiveness likely explains divergent metabolic trajectories during weight gain.

Together, the data summarized in Fig. 1 and Tables 1 and 2 support a unifying model in which metabolic health is governed not by adiposity itself, but by the precision of lipolytic control. Obesity may remain metabolically benign or become pathogenic depending on whether lipolytic flux remains suppressible, temporally coordinated, and appropriately coupled to lipid storage and oxidation. Framing metabolic disease around the quality of adipose regulation rather than fat mass provides a more accurate framework for understanding pathogenesis and for developing therapies that restore metabolic resilience.

## Key References

- Yu X, Chen S, Funcke J-B, Straub LG, Pirro V, Emont MP, et al. The GIP receptor activates futile calcium cycling in white adipose tissue to increase energy expenditure and drive weight loss in mice. Cell Metabolism. 2025;37(1):187–204.e7.10.1016/j.cmet.2024.11.003.
  - ○ This study identifies a novel mechanism by which adipocyte GIP receptor signaling increases energy expenditure through futile calcium cycling in white adipose tissue. By linking incretin signaling directly to adipocyte metabolic activity and lipid turnover, the work provides mechanistic insight into how incretin-based therapies promote weight loss and metabolic improvement.
- Regmi A, Aihara E, Christe ME, Varga G, Beyer TP, Ruan X, et al. Tirzepatide modulates the regulation of adipocyte nutrient metabolism through long-acting activation of the GIP receptor. Cell Metab. 2024;36(7):1534–49.e7.10.1016/j.cmet.2024.05.010.
  - ○ This study shows that tirzepatide exerts sustained effects on adipocyte nutrient metabolism through prolonged activation of the GIP receptor. The findings provide mechanistic evidence that incretin-based therapies influence adipocyte lipolytic regulation, linking pharmacologic weight-loss signaling to the control of fatty-acid flux in adipose tissue.
- Andersson DP, Sørensen TIA, Arner P. The Anti-Lipolytic Effect of Insulin in Adipocytes Associates with the Magnitude of Dietary Induced Loss in Body Weight and Fat Mass: A Longitudinal Study. Obesity Facts. 2025.10.1159/000547949.
  - ○ This study demonstrates that greater insulin-mediated suppression of adipocyte lipolysis predicts larger diet-induced reductions in body weight and fat mass. The findings support the concept that effective control of lipolytic flux is a determinant of successful weight loss and metabolic regulation.
- Yeh Y-S, Evans TD, Iwase M, Jeong S-J, Zhang X, Liu Z, et al. Identification of lysosomal lipolysis as an essential noncanonical mediator of adipocyte fasting and cold-induced lipolysis. The Journal of Clinical Investigation. 2025;135(6).10.1172/JCI185340.
  - ○ This paper reveals lysosomal lipolysis as a previously underappreciated pathway contributing to adipocyte lipid mobilization during fasting and cold exposure. The findings expand the canonical ATGL–HSL framework and demonstrate that multiple lipolytic systems coordinate adipocyte lipid turnover.

## Data Availability

There is no research data outside the submitted manuscript file.

## Declarations

### Third Party Material

All of the material is owned by the authors and/or no permissions are required.
